# Standardized uptake value of ^18^F-fluorodeoxyglucose positron emission tomography for prediction of tumor recurrence in breast cancer beyond tumor burden

**DOI:** 10.1186/s13058-014-0502-y

**Published:** 2014-12-31

**Authors:** Sung Gwe Ahn, Jong Tae Park, Hak Min Lee, Hak Woo Lee, Tae Joo Jeon, Kyunghwa Han, Seung Ah Lee, Seung Myung Dong, Young Hoon Ryu, Eun Ju Son, Joon Jeong

**Affiliations:** 1 Department of Surgery, Gangnam Medical Research Center, Yonsei University College of Medicine, Seoul, Korea; 2 Department of Nuclear Medicine, Gangnam Medical Research Center, Yonsei University College of Medicine, Seoul, Korea; 3 Biostatistics Collaboration Unit, Gangnam Medical Research Center, Yonsei University College of Medicine, Seoul, Korea; 4 Department of Surgery, Eulji University College of Medicine, Seoul, Republic of Korea; 5 Research Institute and Hospital, National Cancer Center, Goyang, Gyeonggi Republic of Korea; 6 Department of Radiology, Gangnam Severance Hospital, Yonsei University College of Medicine, Seoul, Korea

## Abstract

**Introduction:**

^18^F-fluorodeoxyglucose positron emission tomography (FDG-PET) can reveal the metabolic activity of malignant tumors. Recent advances gained from molecular studies suggest that tumor biology can be a good predictor of prognosis in breast cancer. We compared the ability of maximum standardized uptake values (SUV_max_) derived by FDG-PET with tumor burden in predicting tumor recurrence for patients with breast cancer.

**Methods:**

496 patients with breast cancer who underwent preoperative FDG-PET between April 2004 and May 2009 were retrospectively identified. SUV_max_ was obtained by FDG-PET, and the cutoff point was defined using a time-dependent receiver operating characteristic curve for recurrence-free survival (RFS). The primary endpoint was RFS.

**Results:**

In multivariate analysis for RFS, SUV_max_ carried independent prognostic significance (hazard ratio, 2.39; 95% confidence interval, 1.20 to 4.76; *P* = 0.012). When the patients were classified into four groups according to the combined factors of tumor size (≤2 cm versus >2 cm) and SUV_max_ (<4 versus ≥4), RFS differed significantly (*P* < 0.001). Similarly, SUV_max_ had prognostic value in combination with nodal status (negative versus positive) or stage (I versus II and III) (*P* < 0.001 and *P* = 0.001, respectively). In hormone receptor–positive disease, SUV_max_ remained a significant prognostic factor for RFS based on multivariate analysis.

**Conclusions:**

Our results highlight the prognostic value of FDG-PET in prediction of tumor relapse for patients with breast cancer. Particularly in patients with hormone receptor–positive disease, the tumor metabolic information provided by FDG-PET is more significantly correlated with prognosis than tumor burden.

**Electronic supplementary material:**

The online version of this article (doi:10.1186/s13058-014-0502-y) contains supplementary material, which is available to authorized users.

## Introduction

Tumor burden, represented by tumor size and the number of involved lymph nodes, is the most important prognostic factor for breast cancer recurrence [1],[2] because advanced-stage tumors are more likely to have distant metastases. In the genomics era, rapid advances in translational research have greatly improved our understanding of breast cancer biology. This work provides us with the tools that can identify intrinsic subtypes of breast cancer and discriminate a prognosis according to subtype [3], highlighting the clinical availability of tumor biology in breast cancer prognosis [4],[5]. These studies provide evidence that small tumors with undesirable biology can lead to a worse prognosis than large tumors with favorable biology. Therefore, to deliver more effective personalized medical treatment to individual patients, there is an increasing need to evaluate cancer with tumor biology integration, as well as simple anatomical staging.

^18^F-fluorodexoyglucose positron emission tomography (FDG-PET) is a useful tool in the prediction of tumor recurrence, as well as for providing relevant anatomical information, because this imaging modality reflects tumor biology well [6],[7]. It is one of the new tools that can capture tumor biology without an invasive procedure. The degree of FDG uptake reflects the metabolic characteristics of tumors and can be used as a prognostic factor in various malignancies. In breast cancer, studies have shown the contribution of tumor biology to increased FDG uptake [8]-[10] and have demonstrated that FDG uptake is associated with aggressive tumor characteristics [11],[12].

As like other molecular markers were compared or integrated with tumor burden, we wondered whether the prognostic power of current clinical parameters improves when the biologic information of FDG-PET is combined with them. In this retrospective study, we evaluated the potential of FDG uptake as a prognostic indicator in breast cancer as compared to, and in combination with, tumor burden.

## Methods

### Patient selection

**Figure 1:**
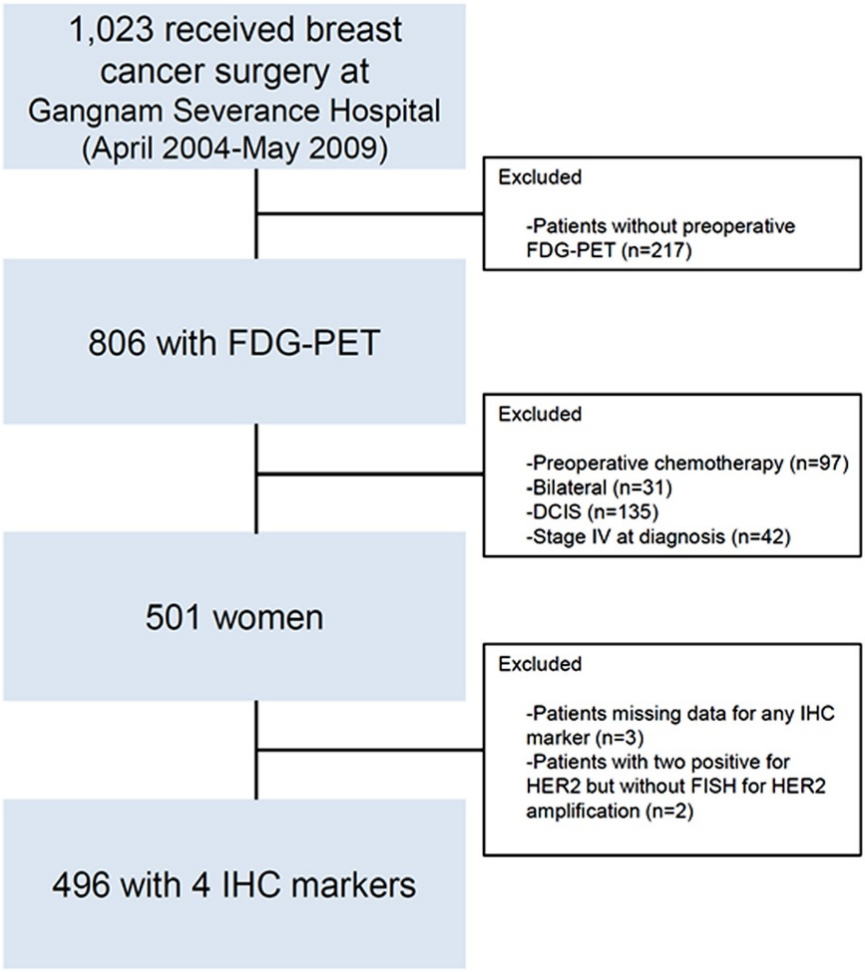
**CONSORT chart outlining the study plan.** DCIS, Ductal carcinoma *in situ*; FDG-PET, ^18^F-fluorodeoxyglucose positron emission tomography; FISH, Fluorescence *in situ* hybridization; HER2, Human epidermal growth factor receptor 2; IHC Immunohistochemistry.

Between April 2004 and May 2009, 1,053 women consecutively underwent surgery for breast cancer at our institution. Of these 1,053 patients, 835 underwent preoperative FDG-PET as a part of their routine preoperative staging. Patients were excluded on the basis of the following criteria: known bilateral breast cancer (*n* = 31), preoperative chemotherapy (because chemotherapy can affect tumor characteristics related to FDG uptake) (*n* = 94), ductal carcinoma *in situ* (*n* = 135) and distant metastases at initial assessment (*n* = 42). Among these patients, 501 women of interest were identified. Patients missing data for any immunohistochemical marker were excluded (*n* = 3). Patients with an immunohistochemistry (IHC) scores of 2+ for human epidermal growth factor receptor 2 (HER2), but without fluorescence *in situ* hybridization (FISH) results for HER2 amplification, were also excluded (*n* = 2). Data for the remaining 496 patients were entered into the analysis (Figure 1).

For the immunohistochemical study of four markers, formalin-fixed, paraffin-embedded tissue sections obtained from the surgical specimens were stained with appropriate antibodies for estrogen receptor (ER) (Novocastra; Leica Microsystems, Newcastle upon Tyne, UK), progesterone receptor (PR) (Novocastra; Leica Microsystems), HER2 (Ventana Medical Systems, Tucson, AZ, USA) and Ki-67 (MIB-1; Dako, Glostrup, Denmark). For HER2 evaluation, membranous staining was graded with a score of 0, 1+, 2+ or 3+ [13]. HER2 status was considered positive with a score of 3+ and negative with a score of 0 or 1+. Tumors with a score of 2+ were sent for FISH testing performed using the PathVysion HER-2 DNA Probe Kit (Abbott Molecular, Des Plaines, IL, USA).

The staging was performed according to the American Joint Committee on Cancer (AJCC) system [14]. The Elston-Ellis modification of the Scarff-Bloom-Richardson grading system was used for tumor grading. Adjuvant systemic therapy and/or radiotherapy were administered according to the standard guidelines based on patient age, primary tumor characteristics and axillary lymph node status. Endocrine therapy was given to patients whose tumors were positive for hormone receptor expression. The follow-up protocol included planned regular visits every 6 months, and missed appointments were followed by telephone calls to minimize the number of patients lost to follow-up and improve the accuracy of the survival data. The final update to the clinical database was made in December 2013.

The institutional review board (IRB) of Gangnam Severance Hospital, Yonsei University, Seoul, Korea, approved the study in accordance with Good Clinical Practice guidelines and the Declaration of Helsinki. The IRB granted a waiver of written documentation of informed consent from all participants because of the retrospective study design.

### ^18^F-fluorodeoxyglucose positron emission tomography

Prior to undergoing FDG-PET, each patient was asked to fast for a minimum of 8 hours, and blood glucose levels were controlled to <130 mg/dl. Patients received an intravenous injection of ^18^F-FDG (0.14 MBq) in the arm contralateral to the primary tumor. Sixty minutes after injection of ^18^F-FDG, whole-body emission scans were obtained using a Philips Allegro PET camera (Philips Medical Systems, Cleveland, OH, USA). Scans were obtained with the patient in the supine position with the arms raised. Attenuation-corrected transaxial images were reconstructed with an iterative transmission algorithm (row-action maximum likelihood three-dimensional protocol) using a three-dimensional image filter in a 128 × 128 matrix. For semiquantitative evaluations, maximum standardized uptake value (SUV_max_) was calculated by measuring the ^18^F-FDG absorption by tumors in the region of interest (ROI) using the following equation: SUV_max_ = (maximal radioactivity concentration in the ROI (μCi/g)/injected dose (μCi)/patient’s weight (kg)). All FDG-PET scans were reviewed by two nuclear medicine radiologists who were blinded to survival data. SUV_max_ was obtained at the time of the imaging procedure.

### Statistical analysis

The cutoff point of SUV_max_ was obtained by using the time-dependent receiver operating characteristic (ROC) curve. Age is presented in the study as median value with a range and was compared by the Mann-Whitney *U* test. Discrete variables were compared by performing a χ^2^ test. The primary endpoint was recurrence-free survival (RFS), which was measured from the date of the first curative surgery to the date of the first tumor recurrence, including locoregional recurrence or distant metastasis or death. Breast cancer–specific survival (BCSS) was measured from the date of the first curative surgery to the date of the last follow-up or until death due to breast cancer during the follow-up period. The Kaplan-Meier method was utilized to estimate RFS or BCSS. Using Harrell c-statistics [15], the concordance index (*c*-index) was calculated to measure the concordance for time-to event data, in which increasing values between 0.5 and 1.0 indicated improved prediction. The significant prognostic factors associated with RFS were selected based on the *c*-index (Additional file 1). The Cox proportional hazards regression model was used for multivariable survival analysis. To assess the additional prognostic value of SUV_max_, we used changes in the likelihood ratio values (LR − Δχ^2^) to quantitatively measure the relative amount of information for SUV_max_ compared to the model without SUV_max_. The cutoff value of young age was defined as 35 years in accordance with a previous Korean study [16]. SPSS version 18 (SPSS, Chicago, IL, USA) and R [17] were used to perform these analyses. Statistical significance was defined by a *P*-value <0.05 or a 95% confidence interval (CI).

## Results

### Definition of cutoff point for maximum standardized uptake value

The cutoff point of SUV_max_ was obtained using the time-dependent ROC. The time-dependent ROC curve for SUV_max_ in relation to RFS yielded an area under the curve of 0.673 (95% CI, 0.588 to 0.753) (Additional file 2). Youden’s index was the highest for SUV_max_ of 4.2. Considering the clinical application, we defined the SUV_max_ cutoff as 4.

### Patient characteristics

A total of 496 patients with breast cancer were included in the analysis. The median age of the cohort was 48 years (range, 25-80 years). The median and mean SUV_max_ were 4.3 ± 3.1 and 3.2 (range, 0.3-32.9), respectively. When patients were divided into two groups according to SUV_max_, these groups differed significantly in T stage, N stage, AJCC stage, which represent tumor burden. They also differed in characteristics reflecting tumor biology, including histologic grade, ER, PR, HER2, and Ki67. In considering the distribution of tumor subtypes, the group with high SUV_max_ had a higher rate of luminal B, HER2, and triple-negative subtypes. In contrast, the proportion of patients with the luminal A subtype was relatively low in the group with high SUV_max_ (Table 1). A higher rate of mastectomy was noted in the group with high SUV_max_ (Table 1).

**Table 1:** **Baseline characteristics according to maximum standardized uptake values** ^**a**^

Characteristics		All patients	High SUV	Low SUV	***P*** -value ^b^
**Age at diagnosis, yr**					0.698
	Median (range)	48 (25 to 80)	48 (25 to 79)	49 (28 to 80)	
**Histology**					<0.001
	Invasive ductal carcinoma	416 (83.9)	173 (87.8)	243 (81.3)	
	Invasive lobular carcinoma	22 (4.4)	1 (0.5)	21 (7.0)	
	Mucinous carcinoma	13 (2.6)	2 (1.0)	11 (3.7)	
	Tubular carcinoma	6 (1.2)	0 (0.0)	6 (2.0)	
	Medullary carcinoma	4 (0.8)	4 (0.8)	0 (0.0)	
	Other invasive carcinoma	35 (7.7)	17 (8.6)	18 (6.0)	
**T classification**					<0.001
	T1	270 (54.4)	68 (34.5)	202 (67.6)	
	T2	217 (43.8)	126 (64.0)	91 (30.4)	
	T3	9 (1.8)	3 (1.5)	6 (2.0)	
**N classification**					0.016
	N0	329 (66.3)	115 (58.4)	214 (71.6)	
	N1	123 (24.8)	59 (29.9)	64 (21.4)	
	N2	30 (6.0)	17 (8.6)	13 (4.3)	
	N3	14 (2.8)	6 (3.0)	8 (2.7)	
**AJCC stage**					<0.001
	I	200 (40.3)	42 (21.3)	158 (52.8)	
	II	252 (50.8)	131 (66.5)	121 (40.5)	
	III	44 (8.9)	24 (12.2)	20 (6.7)	
**Histologic grade** ^**c**^					<0.001
	1	157 (35.0)	43 (22.8)	114 (44.0)	
	2	199 (44.4)	78 (41.3)	121 (46.7)	
	3	92 (20.5)	68 (36.0)	24 (9.3)	
**ER**					0.001
	Positive	304 (61.3)	102 (51.8)	202 (67.6)	
	Negative	192 (38.7)	95 (48.2)	97 (32.4)	
**PR**					0.005
	Positive	293 (59.1)	97 (49.2)	196 (65.6)	
	Negative	203 (40.9)	100 (50.8)	103 (34.4)	
**HER-2** ^**d**^					<0.001
	Positive	127 (25.6)	72 (36.5)	55 (18.4)	
	Negative	369 (74.4)	125 (63.5)	244 (81.6)	
**Ki67**					<0.001
	High	102 (20.6)	64 (32.5)	38 (12.7)	
	Low	394 (79.4)	133(67.5)	261 (87.3)	
**Subtypes**					<0.001
	Luminal A	257 (51.8)	71 (36.0)	186 (62.2)	
	Luminal B	71 (14.4)	39 (19.8)	32 (10.7)	
	HER2	83 (16.7)	45 (22.8)	38 (12.7)	
	Triple negative	85 (17.1)	42 (21.3)	43 (14.4)	
**Surgery type**					0.043
	Mastectomy	352 (70.9)	150 (76.1)	202 (67.5)	
	Breast-conserving surgery	144 (29.1)	47 (24.9)	97 (32.5)	
**Adjuvant chemotherapy**					<0.001
	Yes	347 (70.0)	162 (82.2)	185 (61.9)	
	No	149 (30.0)	35 (17.8)	114 (38.1)	
**Adjuvant endocrine therapy**					0.001
	Yes	332 (66.9)	114 (57.9)	218 (72.9)	
	No	164 (33.1)	83 (42.1)	81 (27.1)	
**Adjuvant radiotherapy**					0.915
	Yes	189 (38.1)	74 (37.6)	115 (38.5)	
	No	307 (61.9)	123 (62.4)	184 (61.5)	

^a^AJCC, American Joint Committee on Cancer; ER, Estrogen receptor; HER2, Human epidermal growth factor receptor 2; PR, Progesterone receptor; SUV_max_, Maximum standardized uptake value. Data are number of patients (%), except for age. ^b^χ^2^ test. ^c^Data with missing values. ^d^HER2 positivity was defined as a 3+ score on immunohistochemistry or amplification on fluorescence *in situ* hybridization.

### Survival outcome

**Figure 2:**
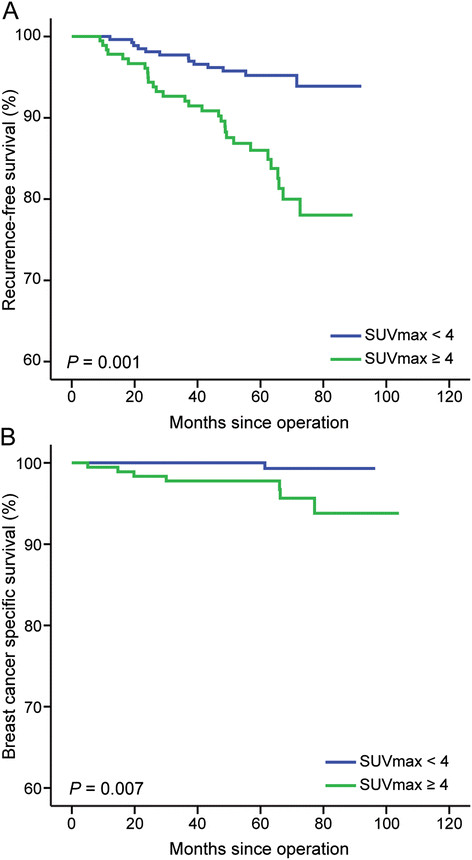
**Kaplan-Meier plots for disease-free survival and breast cancer–specific survival. (A)** Recurrence-free survival (*P* = 0.001). **(B)** Breast cancer–specific survival (*P* = 0.007). SUV_max_, Maximum standardized uptake value. All *P*-values were calculated by the log-rank test.

At a median follow-up of 6.03 years, tumors had recurred in 40 patients. There were 13 patients with locoregional recurrences, 25 with distant metastases and 2 with combined local recurrence and distant metastases. During the follow-up period, 11 deaths occurred, 8 of which were breast cancer–specific and 3 of which were not breast cancer–specific. The probability of RFS at 6 years was 95.6% for patients with low SUV_max_ and 86.8% for patients with high SUV_max_. High SUV_max_ was significantly predictive of decreased RFS (*P* < 0.001 by log-rank test) (Figure 2A). Furthermore, patients with high SUV_max_ showed a reduced BCSS (*P* = 0.007 by log-rank test) (Figure 2B). When adjusted for age of diagnosis, T stage, nodal status and ER status using the Cox proportional hazards regression model, high SUV_max_ was significantly associated with risk of tumor relapse (hazard ratio, 2.39, 95% CI, 1.20 to 4.76) (Table 2). For this model, the Harrell *c*-index was 0.745. The *c*-index for the multivariate model without SUV_max_ was 0.724. The LR-Δχ^2^ showed a significant improvement of the additional prognostic utility of SUV_max_.

**Table 2:** **Multivariate analysis for recurrence-free survival using Cox proportional hazards regression model**

Factors		Hazard ratio	95% CI	***P*** -value
**Age**				0.144
	>35 yr	Reference		
	≤35 yr	1.86	0.81 to 4.25	
**Tumor size**				0.151
	≤2 cm	Reference		
	>2 cm	1.63	0.84 to 3.19	
**Nodal status**				0.038
	Negative	Reference		
	Positive	1.93	1.04 to 3.59	
**Estrogen receptor status**				0.021
	Positive	Reference		
	Negative	2.19	1.12 to 4.27	
**HER2 status**				0.389
	Negative	Reference		
	Positive	1.33	0.69 to 2.57	
**SUV**_**max**_^**b**^				0.013
	Low (<4)	Reference		
	High (≥4)	2.39	1.20 to 4.76	

^a^CI, Confidence interval; HER2, Human epidermal growth factor receptor-2; SUV_max_, Maximum standardized uptake value. ^b^*P* = 0.009 and χ^2^ = 25.41 for the comparison with the analysis without SUV_max_ (by the likelihood ratio test).

### Prognostic value of a combined maximum standardized uptake values with tumor burden

**Figure 3:**
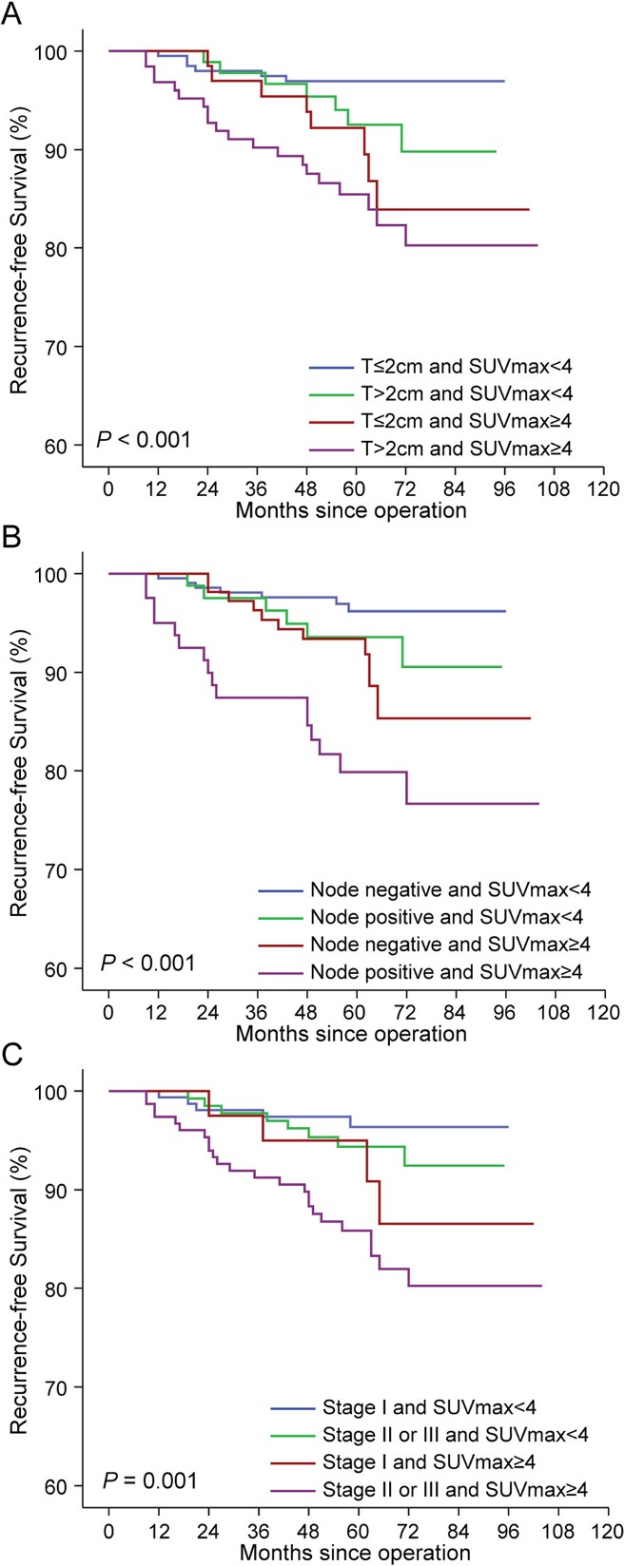
**Kaplan-Meier plots for recurrence-free survival according to combined factors with tumor burden and SUV**_**max**_**. (A)** Tumor size (*P* < 0.001) **(B)** Node status (*P* < 0.001) **(C)** Stage (*P* = 0.001). SUV_max_, Maximum standardized uptake value. All *P*-values were calculated by the log-rank test.

Four patient groups were classified according to SUV_max_ and tumor size: (1) tumor size ≤2 cm and SUV_max_ <4; (2) tumor size >2 cm and SUV_max_ <4; (3) tumor size ≤2 cm and SUV_max_ ≥4; and (4) tumor size >2 cm and SUV_max_ ≥4. The RFS of the four groups differed significantly (*P* < 0.001) (Figure 3A). Within the groups of large tumor size (>2 cm) and small tumor size (≤2 cm), RFS differed significantly according to the SUV_max_ (*P* = 0.049 and *P* = 0.009, respectively). Conversely, within the groups of high SUV_max_ and low SUV_max_, RFS did not differ according to tumor size (*P* = 0.350 and *P* = 0.096, respectively).

Furthermore, SUV_max_ was significantly predictive of RFS in combination with nodal status (*P* < 0.001) (Figure 3B). Node-positive patients with high SUV_max_ had worse outcomes, whereas node-negative patients with low SUV_max_ had better outcomes. Similarly, SUV_max_ combined with stage was significantly correlated with RFS (*P* = 0.001) (Figure 3C).

### Maximum standardized uptake values in luminal breast cancer

After the patients were divided into three subtypes (luminal, HER2 or triple-negative), multivariate analysis for RFS was performed in each subtype. In luminal subtypes, which were defined as hormone receptor–positive breast cancer (ER-positive and/or PR-positive), SUV_max_ was found to be a significant prognostic factor for RFS based on multivariate analysis (Table 3). However, in HER2 or triple-negative subtypes, SUV_max_ was not an independent prognostic factor (Additional file 3).

**Table 3:** **Multivariate analysis for recurrence-free survival using Cox proportional hazards regression model in hormone receptor–positive disease** ^**a**^

Factors		Hazard ratio	95% CI	***P*** -value
**Age**				0.001
	>35 yr	Reference		
	≤35 yr	6.61	2.23 to 19.57	
**Tumor size**				0.706
	≤2 cm	Reference		
	>2 cm	0.815	0.28 to 2.35	
**Nodal status**				0.451
	Negative	Reference		
	Positive	1.49	0.53 to 4.21	
**HER2 status**				0.277
	Negative	Reference		
	Positive	1.87	0.61 to 5.77	
**SUV**_**max**_				0.033
	Low (<4)	Reference		
	High (≥4)	3.56	1.11 to 11.41	

^a^CI, Confidence interval; HER2, Human epidermal growth factor receptor 2; SUV_max_, Maximum standardized uptake value.

**Figure 4:**
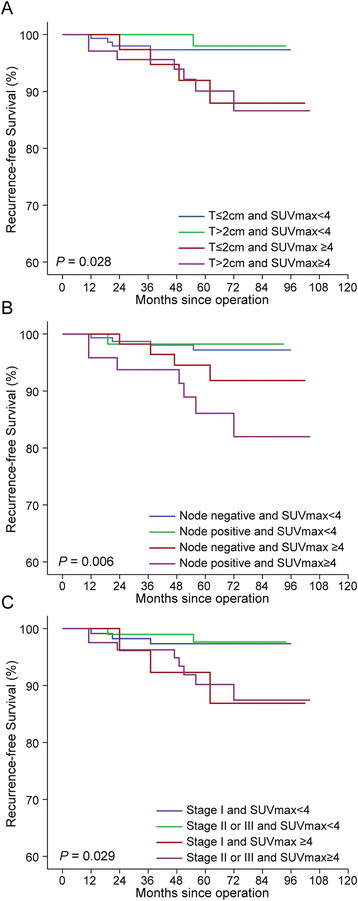
**Kaplan-Meier plots for recurrence-free survival according to a combined factor that includes both tumor burden and SUV**_**max**_**in hormone receptor-positive cancer. (A)** Tumor size (*P* = 0.028) **(B)** Node status (*P* = 0.006) **(C)** Stage (*P* = 0.029). SUV_max_, Maximum standardized uptake value. All *P*-values were calculated by the log-rank test.

The prognostic value of SUV_max_ combined with tumor burden was also assessed in hormone receptor–positive breast cancer. When the patients were classified into four groups according to both combined factors, RFS differed significantly (*P* < 0.001) (Figure 4A). There was no difference in RFS when patients were stratified by tumor size within the groups with high SUV_max_ and low SUV_max_ (*P* = 0.950 and *P* = 0.688, respectively). However, within the groups with small tumor sizes (≤2 cm), a significantly reduced RFS was found in patients with high SUV_max_ (*P* = 0.044). In patients with large tumor sizes (>2 cm), RFS did not differ significantly according to SUV_max_ (*P* = 0.065), possibly due to the limited number of patients (*n* = 122).

In luminal breast cancer, SUV_max_ was still predictive of RFS in combination with nodal status (negative vs. positive) and stage (I vs. II and III) (*P* < 0.006 and *P* = 0.029, respectively) (Figure 4B and 4C).

## Discussion

The results of our study demonstrate the ability of SUV_max_ to predict clinical outcomes in a large cohort of breast cancer patients who underwent FDG-PET. SUV_max_ carried independent prognostic significance in multivariate analysis for prediction of tumor relapse. Attempts to validate FDG uptake as a prognostic indicator in breast cancer have been made in previous studies [18]-[20]. However, failure to be validated as an independent prognostic factor [18], small number of patients [19] and analysis based on a web-accessible risk-assessment model (Adjuvant! Online) [20] were limitations. Despite these limitations, those studies provided evidence that that FDG uptake has potential as a prognostic marker in breast cancer, which seems reasonable because tumors with increased glucose uptake show aggressive tumor behaviors and high proliferative propensities [8]-[10]. Other studies have consistently shown that breast cancer with a high SUV_max_ is associated with ER negativity, high histologic grade, high Ki67 and the triple-negative subtype [10]-[12], which is consistent with our data (Table 1). In support of the clinical significance of tumor biology–associated glucose metabolism are recent studies showing that several signaling pathways implicated in cell proliferation and tumor progression also regulate metabolic pathways [21]-[24].

Particularly in the survival analyses using a combined factor with SUV_max_ and tumor burden, SUV_max_ showed a superior prediction of RFS in breast cancer compared with clinical tumor load. After four groups were formed using SUV_max_ and tumor size, within the groups with high or low SUV_max_, tumor size did not provide additional prognostic differentiation (Figure 3A). However, within the groups with large or small tumor size, SUV_max_ improved the prediction of RFS. Similar results were seen when SUV_max_ was combined with nodal status or AJCC stage (Figures 3B and 3C). These findings suggest that when tumor biology is considered in addition to clinical tumor burden, prediction of breast cancer prognosis can be improved. SUV_max_ could provide powerful prognostic information about tumor relapse that is superior to considering only tumor burden, similar to the contribution of molecular subtype.

There are established molecular predictors reflecting tumor biology and predicting prognosis in breast cancer. Although the reason that the multigene assays are actively utilized for ER-positive disease has not been fully clarified, authors of meta-analyses of various multigene breast cancer signatures concluded that the prognostic values of the signatures are comparable when evaluated in hormone receptor–positive breast cancers, presumably due to the fact that the proliferation modules within these diverse gene signatures are a common driving force behind their overall prognostic performance [25],[26]. By contrast, hormone receptor–negative breast cancers are more proliferative and are usually classified as high risk or are not the appropriate target population for these prognostic signatures [25],[26]. In the same context, our results show that the prognostic significance of SUV_max_ is distinct for luminal tumors (Table 3, Figure 4).

Furthermore, the mean SUV_max_ for the luminal subtype was the lowest, whereas the values for the HER2 and triple-negative subtypes were comparatively higher (Additional file 4). This finding is concordant with previous reports comparing SUV_max_ between the IHC-defined subtypes [27]. It seems reasonable that HER2-positive or triple-negative tumors would show increased accumulation of FDG, because these tumors have an aggressive phenotype and are associated with a high rate of proliferation, high Ki67 concentration and high histologic grades. These associations between aggressive markers and high SUV_max_ were concordantly observed in our study (Table 1). Because HER2-positive or triple-negative tumors generally show high SUV_max_, this may also lead to a reduced prognostic significance of SUV_max_ in these kinds of tumors.

We acknowledge several limitations inherent in our study’s retrospective design. We were unable to control for variations in adjuvant therapy that may have influenced survival outcomes. Compared to the low SUV_max_ group, the patients in the high SUV_max_ group received more chemotherapy and less endocrine therapy, likely because they had more advanced stage disease and ER negativity. The cutoff point for SUV_max_ defined within a single cohort also needs to be validated in an external cohort. However, there was not a significant difference in the number of patients who received radiation treatment between the high SUV_max_ group and the low SUV_max_ group. There was also no survival difference between patients who received adjuvant chemotherapy or radiotherapy (Additional file 1).

## Conclusions

Our study highlights the prognostic value of FDG-PET in predicting tumor relapse for breast cancer patients. We provide evidence supporting the potential utility of FDG-PET in combination with clinical tumor burden for the assessment of prognosis as well as evaluation of tumor location in patients with breast cancer. These results lay the groundwork for future studies on the prognostic implication of SUV_max_ for breast cancer treatment.

## Additional files

## Electronic supplementary material

Additional file 1: **Details of our process for selecting variables and optimizing the multivariate model based on** ***c*** **-index.** (DOCX 112 KB)

Additional file 2: **Defined the cutoff value of SUV**_**max**_**.** (DOCX 54 KB)

Additional file 3: 1. Multivariate analysis for recurrence-free survival using the Cox proportional hazards regression model in HER2-positive disease or triple-negative disease. 2. Regimens for adjuvant chemotherapy used in our patients. (DOCX 21 KB)

Additional file 4: **SUV**_**max**_**according to the intrinsic subtypes.** (DOCX 26 KB)

Below are the links to the authors’ original submitted files for images.

Authors’ original file for figure 1

Authors’ original file for figure 2

Authors’ original file for figure 3

Authors’ original file for figure 4

## Abbreviations

AJCC: American Joint Committee on Cancer
BCSS: Breast cancer–specific survival
CI: Confidence interval
*c*-index: Concordance index
ER: Estrogen receptor
FDG-PET: ^18^F-fluorodexoyglucose positron emission tomography
FISH: Fluorescence *in situ* hybridization
HER2: Human epidermal growth factor receptor 2
HR: Hazard ratio
IHC: Immunohistochemistry
IRB: Institutional review board
PR: Progesterone receptor
RFS: Recurrence-free survival
ROC: Receiver operating characteristic
ROI: Region of interest
SUV_max_: Maximum standardized uptake value
