# Supplementary material for: Standardized uptake value of 18F-fluorodeoxyglucose positron emission tomography for prediction of tumor recurrence in breast cancer beyond tumor burden
Source: Breast Cancer Res. 2014 Dec 31;16:502. doi: 10.1186/s13058-014-0502-y (PMC4308858; doi:10.1186/s13058-014-0502-y)
Supplement: Supplementary file 1 — Additional file 1: Details of our process for selecting variables and optimizing the multivariate model based on c -index. (DOCX 112 KB) [file 13058_2014_502_MOESM1_ESM.docx]

To determine the variables used in the multivariate model, we first performed univariate analysis with each variable (Supplemental Table 1). Ki67 was excluded as it was not significant in the analyses. Variance inflation factors (VIF) were estimated, with subtype excluded, because VIF for subtype was 42.3 (Supplemental Table 2).

To calculate tumor burden, we used tumor size and nodal status instead of stage because if stage were to be calculated based on size and nodal status, its VIF would be 4.11 (close to 5). To include hormone receptor status, ER was used as a more representative marker of the hormone receptor. To integrate clinical and pathological information into our multivariate model, we analyzed the data by age, tumor size, nodal status, estrogen receptor, and HER2 status.

To optimize the multivariate model, we compared c-indices among the three models: i) addition of SUV_max_ alone; ii) addition of grade alone; and iii) addition of both SUV_max_ and grade. C-indices for these models have been presented below (Supplemental Figure 1). Among them, the model using SUV_max_ alone showed the highest c-index (0.745) and was selected for this study.

To assess the additional prognostic value of SUV_max_, we used two approaches: one, changes in the likelihood ratio values (LR-Δχ2) to quantitatively measure the relative amount of information for SUV_max_ compared to the model without SUV_max_; and two, the concordance index (c-index) measure of concordance for time-to-event data, in which increasing values between 0.5 and 1.0 indicate an improved prediction. These analyses have been presented below (Supplemental Figure 2). This assessment also showed that the model including SUV_max_ provides significantly improved prognostic values for recurrence-free survival in our population.

**Supplemental Table 1. Univariate analyses according to tumor characteristics**

| **Characteristics** | ***P*-value^a^** | **HR^b^** | **95% CI** |
| --- | --- | --- | --- |
| **Age** | **0.039** | **2.30** | **1.02-5.19** |
| ≤35 vs. >35 |  |  |  |
| **T stage** | **0.008** | **2.11** | **1.22-3.66** |
| >2 cm vs. ≤2 cm |  |  |  |
| **Nodal status** | **0.001** | **1.75** | **1.27-2.41** |
| Positive vs. Negative |  |  |  |
| **AJCC stage** | **0.002** | **2.10** | **1.31-3.37** |
| I vs. II vs. III |  |  |  |
| **Histologic grades** | **0.002** | **2.83** | **1.34-5.32** |
| 1 and 2 vs. 3 |  |  |  |
| **Estrogen receptor** | **0.001** | **2.89** | **1.53-5.46** |
| Positive vs. Negative |  |  |  |
| **Progesterone receptor** | **0.003** | **4.91** | **2.40-10.00** |
| Positive vs. Negative |  |  |  |
| **HER-2** | **0.027** | **2.01** | **1.07-3.76** |
| Negative vs. Positive |  |  |  |
| **Ki67** | 0.157 | 1.64 | 0.82-3.28 |
| Low (<15%) vs. High (15%≥) |  |  |  |
| **Subtypes** | **0.001** | **1.57** | **1.23-2.01** |
| Luminal A vs. Luminal B vs. HER2 vs. TNBC |  |  |  |
| **Dichotomized SUV_max_** | **0.001** | **3.40** | **1.76-6.56** |
| Low vs. High |  |  |  |
| **Adjuvant chemotherapy** | 0.268 | 1.34 | 0.67-2.93 |
| Yes or No |  |  |  |
| **Adjuvant radiotherapy** | 0.288 | 1.40 | 0.75-2.63 |
| Yes or No |  |  |  |
| **Adjuvant endocrine therapy** ^c^ | <0.001 | 2.91 | 1.55-5.44 |
| Yes or No |  |  |  |

HR, hazard ratio; HER2, human epidermal growth factor receptor-2; SUV_max_, maximum standardized uptake value.

^a^ Log-rank test

^b^ HR was calculated using a Cox regression hazard model.

^c^ Adjuvant endocrine therapy was not considered in the multivariate model because its use was confined to luminal tumors.

**Supplemental Table 2. Variance inflation factor (VIF) values for each characteristic**

| **Characteristic** | **VIF** | **VIF after exclusion of subtype** |
| --- | --- | --- |
| **Age** | **1.0201** | **1.0172** |
| ≤35 vs. >35 |  |  |
| **T stage** | **1.1858** | **1.1823** |
| >2 cm vs. ≤2 cm |  |  |
| **Nodal status** | **1.0756** | **1.0391** |
| Positive vs. Negative |  |  |
| **AJCC stage** | **3.1232** | **3.1321** |
| I vs. II vs. III |  |  |
| **Histologic grades** | **1.3274** | **1.2821** |
| 1 and 2 vs. 3 |  |  |
| **Estrogen receptor** | **5.5075** | **2.1938** |
| Positive vs. Negative |  |  |
| **Progesterone receptor** | **5.2132** | **2.1490** |
| Positive vs. Negative |  |  |
| **HER-2** | **5.2902** | **1.1680** |
| Negative vs. Positive |  |  |
| **Subtypes** | **42.2879** | **N/A** |
| Luminal A vs. Luminal B vs. HER2 vs. TNBC |  |  |
| **Dichotomized SUV_max_** | **1.3153** | **1.3041** |
| Low vs. High |  |  |

**Supplemental Figure 1. C-indices for the three multivariate models with: i) addition of histologic grade alone ii) addition of histologic grade and SUV_max_ iii) addition of SUV_max_ alone**


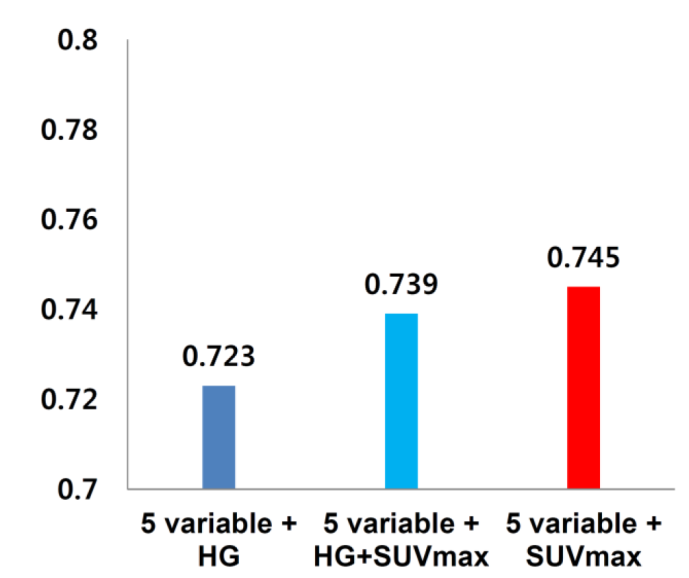


**Supplemental Figure 2. *C*-indices for the multivariate models with or without SUV_max_. Changes in the likelihood ratio values (LR-Δχ^2^) for the quantitative measure of the relative amount of information for SUV_max_ compared to the model without SUV_max_.**

**
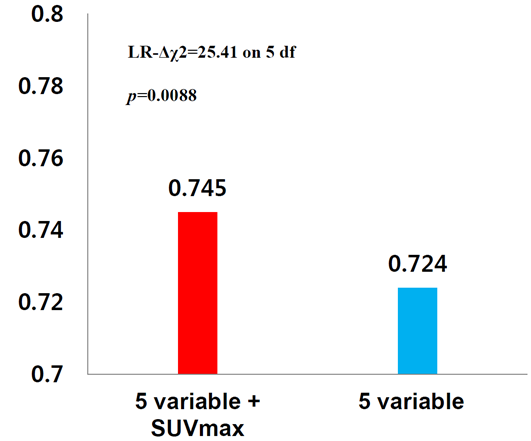
**
