# Supplementary material for: Standardized uptake value of 18F-fluorodeoxyglucose positron emission tomography for prediction of tumor recurrence in breast cancer beyond tumor burden
Source: Breast Cancer Res. 2014 Dec 31;16:502. doi: 10.1186/s13058-014-0502-y (PMC4308858; doi:10.1186/s13058-014-0502-y)
Supplement: Supplementary file 2 — Additional file 2: Defined the cutoff value of SUV max. (DOCX 54 KB) [file 13058_2014_502_MOESM2_ESM.docx]

1. The cut-off point of SUVmax was obtained using the time-dependent ROC. The time-dependent ROC curve for SUVmax in relation to recurrence-free survival yielded the area under the curve of 0.673 (95% CI, 0.588 to 0.753).


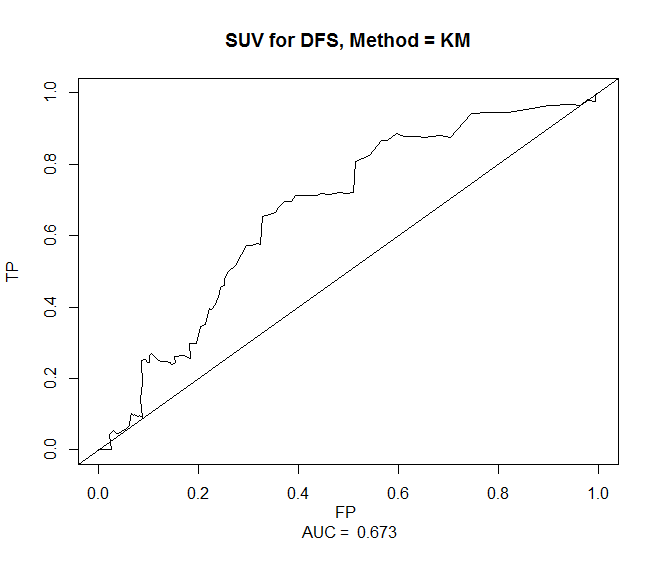


**⇒ AUC=0.673(95%CI: 0.5875, 0.7527)**

2. Youden’s index was the highest for SUVmax of 4.2. Considering the clinical application, we defined the cutoff of SUVmax as 4.

| **SUV** | **Sensitivity** | **1-specificity** | **Youden's index** |
| --- | --- | --- | --- |
|  | 1.0000 | 1.0000 | 0.0000 |
| 0.3 | 0.9981 | 0.9980 | 0.0002 |
| 0.8 | 0.9971 | 0.9935 | 0.0035 |
| 0.9 | 0.9766 | 0.9940 | -0.0173 |
| 1.0 | 0.9792 | 0.9822 | -0.0030 |
| 1.1 | 0.9821 | 0.9773 | 0.0049 |
| 1.2 | 0.9651 | 0.9635 | 0.0015 |
| 1.3 | 0.9685 | 0.9448 | 0.0237 |
| 1.4 | 0.9642 | 0.8997 | 0.0645 |
| 1.5 | 0.9533 | 0.8555 | 0.0978 |
| 1.6 | 0.9460 | 0.8177 | 0.1283 |
| 1.7 | 0.9439 | 0.7791 | 0.1648 |
| 1.8 | 0.9416 | 0.7452 | 0.1964 |
| 1.9 | 0.8759 | 0.7037 | 0.1723 |
| 2.0 | 0.8820 | 0.6846 | 0.1974 |
| 2.1 | 0.8754 | 0.6490 | 0.2265 |
| 2.2 | 0.8768 | 0.6396 | 0.2372 |
| 2.3 | 0.8790 | 0.6120 | 0.2671 |
| 2.4 | 0.8860 | 0.5951 | 0.2909 |
| 2.5 | 0.8660 | 0.5772 | 0.2888 |
| 2.6 | 0.8655 | 0.5658 | 0.2997 |
| 2.7 | 0.8242 | 0.5416 | 0.2826 |
| 2.8 | 0.8083 | 0.5140 | 0.2942 |
| 2.9 | 0.7199 | 0.5097 | 0.2102 |
| 3.0 | 0.7196 | 0.4984 | 0.2212 |
| 3.1 | 0.7203 | 0.4823 | 0.2380 |
| 3.2 | 0.7152 | 0.4624 | 0.2528 |
| 3.3 | 0.7170 | 0.4439 | 0.2731 |
| 3.4 | 0.7124 | 0.4377 | 0.2748 |
| 3.5 | 0.7121 | 0.4080 | 0.3041 |
| 3.6 | 0.7114 | 0.3944 | 0.3169 |
| 3.7 | 0.6963 | 0.3850 | 0.3113 |
| 3.8 | 0.6966 | 0.3713 | 0.3253 |
| 3.9 | 0.6783 | 0.3600 | 0.3183 |
| 4.0 | 0.6640 | 0.3528 | 0.3113 |
| 4.1 | 0.6581 | 0.3421 | 0.3160 |
| 4.2 | 0.6544 | 0.3267 | 0.3277 |
| 4.3 | 0.5762 | 0.3233 | 0.2529 |
| 4.4 | 0.5780 | 0.3185 | 0.2595 |
| 4.5 | 0.5724 | 0.3055 | 0.2669 |
| 4.6 | 0.5738 | 0.2962 | 0.2776 |
| 4.7 | 0.5173 | 0.2740 | 0.2433 |
| 4.8 | 0.4999 | 0.2603 | 0.2396 |
| 4.9 | 0.4786 | 0.2517 | 0.2269 |
| 5.0 | 0.4610 | 0.2518 | 0.2092 |
| 5.1 | 0.4555 | 0.2434 | 0.2121 |
| 5.2 | 0.4392 | 0.2410 | 0.1983 |
| 5.3 | 0.4064 | 0.2339 | 0.1726 |
| 5.4 | 0.3933 | 0.2265 | 0.1668 |
| 5.5 | 0.3953 | 0.2217 | 0.1737 |
| 5.6 | 0.3729 | 0.2178 | 0.1551 |
| 5.7 | 0.3514 | 0.2138 | 0.1376 |
| 5.8 | 0.3465 | 0.2030 | 0.1435 |
| 5.9 | 0.2992 | 0.1956 | 0.1036 |
| 6.1 | 0.2969 | 0.1822 | 0.1147 |
| 6.2 | 0.2571 | 0.1829 | 0.0742 |
| 6.3 | 0.2629 | 0.1707 | 0.0922 |
| 6.4 | 0.2641 | 0.1614 | 0.1027 |
| 6.7 | 0.2618 | 0.1594 | 0.1024 |
| 6.8 | 0.2626 | 0.1525 | 0.1101 |
| 6.9 | 0.2437 | 0.1527 | 0.0910 |
| 7.0 | 0.2413 | 0.1507 | 0.0906 |
| 7.1 | 0.2402 | 0.1463 | 0.0939 |
| 7.2 | 0.2442 | 0.1435 | 0.1008 |
| 7.4 | 0.2475 | 0.1362 | 0.1114 |
| 7.5 | 0.2485 | 0.1338 | 0.1147 |
| 7.6 | 0.2488 | 0.1246 | 0.1242 |
| 7.7 | 0.2462 | 0.1227 | 0.1235 |
| 7.8 | 0.2547 | 0.1147 | 0.1401 |
| 7.9 | 0.2611 | 0.1116 | 0.1495 |
| 8.0 | 0.2682 | 0.1083 | 0.1599 |
| 8.1 | 0.2698 | 0.1059 | 0.1639 |
| 8.2 | 0.2642 | 0.1020 | 0.1622 |
| 8.3 | 0.2454 | 0.1022 | 0.1432 |
| 8.4 | 0.2442 | 0.0978 | 0.1464 |
| 8.5 | 0.2522 | 0.0945 | 0.1577 |
| 8.6 | 0.2492 | 0.0857 | 0.1635 |
| 8.7 | 0.1841 | 0.0875 | 0.0966 |
| 8.8 | 0.1660 | 0.0853 | 0.0807 |
| 8.9 | 0.1413 | 0.0840 | 0.0573 |
| 9.0 | 0.0907 | 0.0884 | 0.0023 |
| 9.1 | 0.0954 | 0.0810 | 0.0144 |
| 9.2 | 0.0935 | 0.0789 | 0.0145 |
| 9.3 | 0.0970 | 0.0762 | 0.0208 |
| 9.5 | 0.0996 | 0.0713 | 0.0283 |
| 9.8 | 0.0973 | 0.0693 | 0.0280 |
| 9.9 | 0.1020 | 0.0664 | 0.0356 |
| 10.0 | 0.0897 | 0.0635 | 0.0262 |
| 10.1 | 0.0669 | 0.0619 | 0.0050 |
| 10.2 | 0.0647 | 0.0599 | 0.0048 |
| 10.5 | 0.0583 | 0.0539 | 0.0043 |
| 10.6 | 0.0561 | 0.0519 | 0.0042 |
| 10.8 | 0.0475 | 0.0439 | 0.0035 |
| 11.2 | 0.0469 | 0.0372 | 0.0097 |
| 11.3 | 0.0444 | 0.0352 | 0.0092 |
| 11.4 | 0.0552 | 0.0292 | 0.0260 |
| 11.5 | 0.0483 | 0.0256 | 0.0228 |
| 12.0 | 0.0449 | 0.0237 | 0.0211 |
| 12.2 | 0.0414 | 0.0219 | 0.0195 |
| 12.4 | 0.0000 | 0.0251 | -0.0251 |
| 12.8 | 0.0000 | 0.0228 | -0.0228 |
| 13.1 | 0.0000 | 0.0205 | -0.0205 |
| 14.5 | 0.0000 | 0.0183 | -0.0183 |
| 14.6 | 0.0000 | 0.0160 | -0.0160 |
| 15.5 | 0.0000 | 0.0137 | -0.0137 |
| 15.6 | 0.0000 | 0.0114 | -0.0114 |
| 15.8 | 0.0000 | 0.0091 | -0.0091 |
| 16.7 | 0.0000 | 0.0068 | -0.0068 |
| 16.8 | 0.0000 | 0.0046 | -0.0046 |
| 19.8 | 0.0000 | 0.0023 | -0.0023 |
| 32.9 | 0.0000 | 0.0000 | 0.0000 |
