# Supplementary material for: Standardized uptake value of 18F-fluorodeoxyglucose positron emission tomography for prediction of tumor recurrence in breast cancer beyond tumor burden
Source: Breast Cancer Res. 2014 Dec 31;16:502. doi: 10.1186/s13058-014-0502-y (PMC4308858; doi:10.1186/s13058-014-0502-y)
Supplement: Supplementary file 3 — Additional file 3: 1. Multivariate analysis for recurrence-free survival using the Cox proportional hazards regression model in HER2-positive disease or triple-negative disease. 2. Regimens for adjuvant chemotherapy used in our patients. (DOCX 21 KB) [file 13058_2014_502_MOESM3_ESM.docx]

**Supplemental Table 1. Multivariate analysis for recurrence-free survival using Cox-regression hazard model in HER2-positive disease**

| **Factors** | **Hazard ratio** | **95% CI** | ***P*-value** |
| --- | --- | --- | --- |
| **Age** |  |  | 1.000 |
| Age > 35 | Reference |  |  |
| Age ≤ 35 | 1.00 | 0.12-8.18 |  |
|  |  |  |  |
| **Tumor size** |  |  | 0.207 |
| T ≤ 2 cm | Reference |  |  |
| T > 2 cm | 2.36 | 0.62-8.97 |  |
|  |  |  |  |
| **Nodal status** |  |  | 0.455 |
| Negative | Reference |  |  |
| Positive | 1.55 | 0.49-4.93 |  |
|  |  |  |  |
| **SUV_max_** |  |  | 0.851 |
| Low (< 4) | Reference |  |  |
| High (≥ 4) | 1.12 | 0.35-3.57 |  |

SUV_max_, maximum standardized uptake value.

**Supplemental Table 2. Multivariate analysis for recurrence-free survival using Cox-regression hazard model in triple-negative disease**

| **Factors** | **Hazard ratio** | **95% CI** | ***P*-value** |
| --- | --- | --- | --- |
| **Age** |  |  | 0.420 |
| Age > 35 | Reference |  |  |
| Age ≤ 35 | 2.33 | 0.30-18.11 |  |
|  |  |  |  |
| **Tumor size** |  |  | 0.331 |
| T ≤ 2 cm | Reference |  |  |
| T > 2 cm | 2.00 | 0.50-8.03 |  |
|  |  |  |  |
| **Nodal status** |  |  | 0.044 |
| Negative | Reference |  |  |
| Positive | 3.26 | 1.04-10.24 |  |
|  |  |  |  |
| **SUV_max_** |  |  |  |
| Low (< 4) | Reference |  | 0.053 |
| High (≥ 4) | 3.74 | 0.98-14.24 |  |

SUV_max_, maximum standardized uptake value.

**Supplemental Table 3. Baseline characteristics according to SUV_max_**

| **Characteristics** | **All patients** | **High SUV** | **Low SUV** | | ***P*-value**^a^ |
| --- | --- | --- | --- | --- | --- |
| **Adjuvant chemotherapy** |  |  |  | 0.839 | |
| CMF | 30 | 13 | 17 |  | |
| AC | 182 | 85 | 97 |  | |
| AC followed by paclitaxel | 135 | 66 | 69 |  | |

AC, Adriamycin-cyclophosphamide; CMF, Cyclophosphamide-Methotrexate-5-Fluorouracil.
