# Supplementary material for: Standardized uptake value of 18F-fluorodeoxyglucose positron emission tomography for prediction of tumor recurrence in breast cancer beyond tumor burden
Source: Breast Cancer Res. 2014 Dec 31;16:502. doi: 10.1186/s13058-014-0502-y (PMC4308858; doi:10.1186/s13058-014-0502-y)
Supplement: Supplementary file 4 — Additional file 4: SUV maxaccording to the intrinsic subtypes. (DOCX 26 KB) [file 13058_2014_502_MOESM4_ESM.docx]

**Supplementary Figure 1. Means of SUV_max_ according to the intrinsic subtypes**


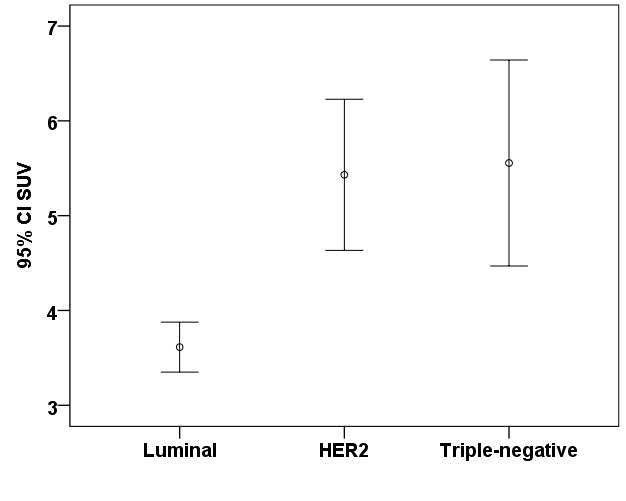


The patients were classified into three intrinsic subtype groups based on four IHC markers: luminal (328, 66%); HER-2 (83, 17%); and triple-negative (85, 17%). One-way ANOVA revealed significant differences in SUV_max_ values between the different subtypes (*P* < 0.001). To adjust for multiple comparisons, pair-wise tests were performed between subtypes using the Bonferroni test. In these comparisons, the mean SUV_max_ for the luminal subtype was the lowest (3.61 ± 2.43), whereas the mean SUV_max_ for the HER-2 (5.43 ± 3.64) and triple-negative subtypes (5.55 ± 5.04) were comparatively higher.

**Supplementary Table 1. Comparison of SUV_max_ according to subtypes**

|  | **Luminal A**  **(n=328)** | **HER2**  **(n=83)** | **Triple Negative**  **(n=85)** | ***P* *** |
| --- | --- | --- | --- | --- |
| **SUV_max_** |  |  |  | < 0.001 |
| Mean | 3.61 | 5.43 | 5.55 |  |
| SD | 2.43 | 3.64 | 5.04 |  |
|  | T†=a | T=b | T=b |  |

SUV_max_, maximum standardized uptake value; SD, standard deviation; HER2, human epidermal growth factor receptor-2.

* *P*-value, One-way ANOVA

†The same letters indicate non-significant difference between groups based on the Bonferroni test
