# Supplementary figures and images for: Standardized uptake value of 18F-fluorodeoxyglucose positron emission tomography for prediction of tumor recurrence in breast cancer beyond tumor burden
Source: Breast Cancer Res. 2014 Dec 31;16:502. doi: 10.1186/s13058-014-0502-y (PMC4308858; doi:10.1186/s13058-014-0502-y)

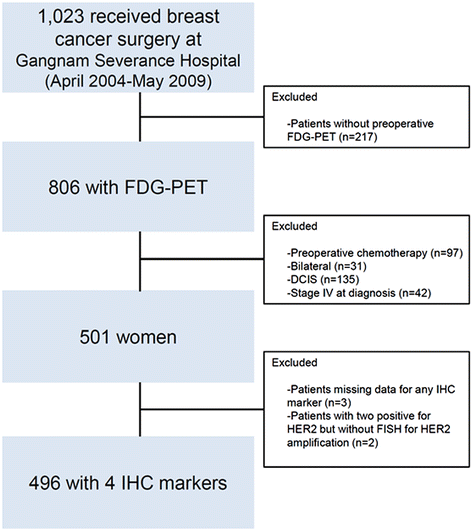

Supplement: Supplementary file 5 — Authors’ original file for figure 1 [file 13058_2014_502_MOESM5_ESM.gif]

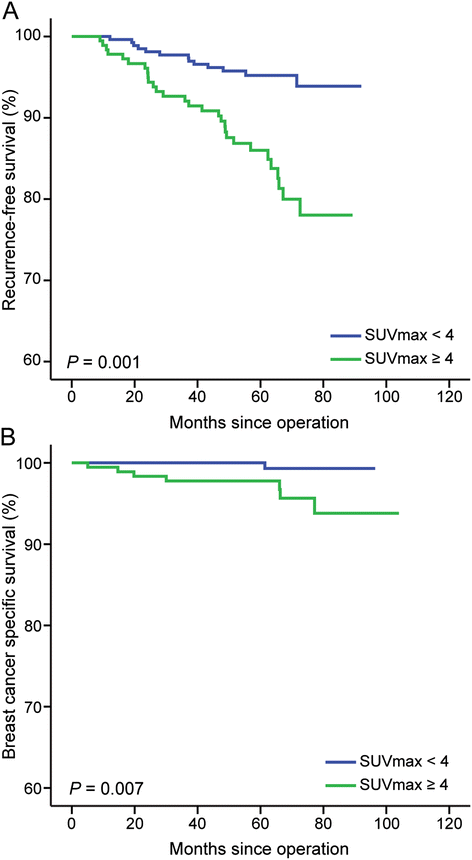

Supplement: Supplementary file 6 — Authors’ original file for figure 2 [file 13058_2014_502_MOESM6_ESM.gif]

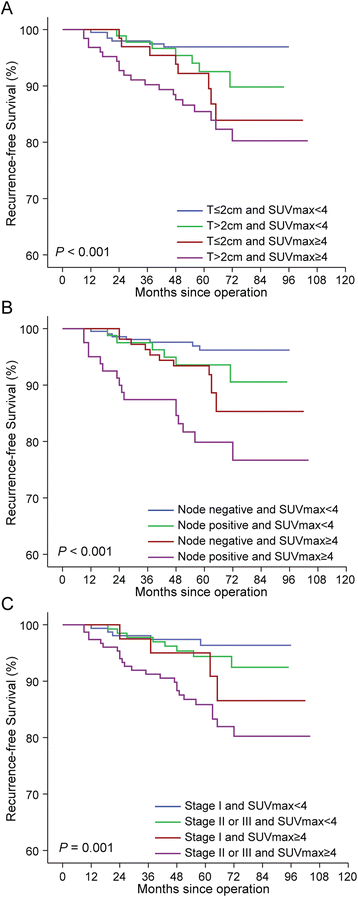

Supplement: Supplementary file 7 — Authors’ original file for figure 3 [file 13058_2014_502_MOESM7_ESM.gif]

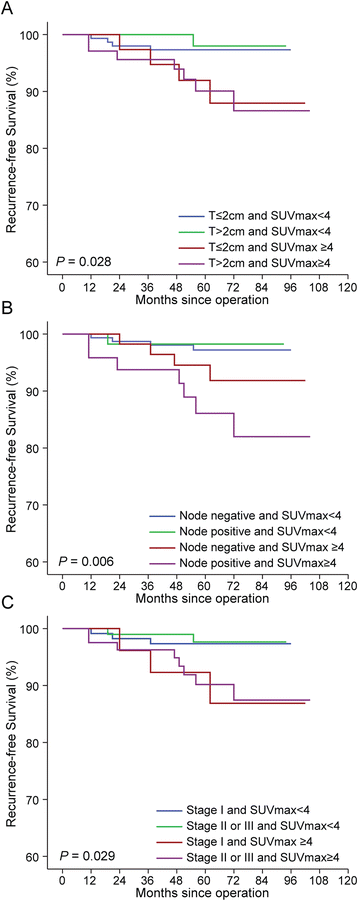

Supplement: Supplementary file 8 — Authors’ original file for figure 4 [file 13058_2014_502_MOESM8_ESM.gif]
